# Physiological responses to salinity increase in blood parrotfish (*Cichlasoma synspilum* ♀ × *Cichlasoma citrinellum* ♂)

**DOI:** 10.1186/s40064-016-2930-x

**Published:** 2016-08-03

**Authors:** Yanming Sui, Xizhi Huang, Hui Kong, Weiqun Lu, Youji Wang

**Affiliations:** 1 College of Fisheries and Life Science, Shanghai Ocean University, 999 Huchenghuan Road, Shanghai, 201306 China; 2 Key Laboratory of Exploration and Utilization of Aquatic Genetic Resources, Ministry of Education, Shanghai, 201306 China; 3 Key Laboratory of East China Sea and Oceanic Fishery Resources Exploitation, Ministry of Agriculture of China, East China Sea Fisheries Research Institute, Chinese Academy of Fisheries Sciences, Shanghai, 20090 China

**Keywords:** Blood parrotfish, Physiological parameter, Salinity

## Abstract

This study aims to evaluate the effects of adding salt to water on the physiological parameters of the blood parrot cichlid (*Cichlasoma synspilum* ♀ × *Cichlasoma citrinellum* ♂). The blood parrot cichlid is a popular species in the aquarium trade because of its behaviour and beauty. Salt is usually added to water during the culture or transportation of this fish. However, the manner by which the fish adjusts its physiological responses to salinity change is unclear. The effects of salinity on serum osmolality, immune-related enzyme activities, Na^+^–K^+^-ATPase activities in the gill, skin carotenoid content and oxygen consumption were analysed. Blood parrotfish individuals were transferred from freshwater to water with four salinity levels (0.16, 2.5, 5 and 7.5 ‰) for 168 h, and physiological responses were evaluated at 0, 6, 12, 24 and 168 h. Results showed no significant differences in serum acid phosphatase and alkaline phosphatase activities, skin carotenoid content and oxygen consumption rate among the different groups. However, the serum osmolality at 6 h was significantly elevated. Moreover, salinity increase stimulated superoxide dismutase (SOD) activity from 0 to 6 h. SOD activity increased from 6 to 24 h but significantly reduced at 168 h when the fish were exposed to salt water. The SOD activity in the salinity 2.5 ‰ group recovered the initial level, whereas those in the salinity 5 and 7.5 ‰ groups decreased to levels lower than the initial level. The gill Na^+^–K^+^-ATPase activity significantly declined with time and salinity increase. Thus, adding an appropriate amount of salt can save energy consumption during osmoregulation and temporarily enhance the antioxidant activity of blood parrotfish. However, this strategy is insufficient for long-term culture. Therefore, adding salt to water only provides short-term benefit to blood parrot cichlid during transportation.

## Background

Salinity is an ecological factor with considerable importance for teleosts. A change in salinity can alter the osmotic pressure between medium and body fluid, causing osmoregulation directly in teleosts. Na^+^–K^+^-ATPase (NKA) is a membrane-spanning enzyme that actively transports Na^+^ out of ionocytes and K^+^ into ionocytes; this enzyme maintains osmotic equilibrium by providing a driving force for other ion-transporting systems (Marshall and Bryson 1998; Hirose et al. 2003; Hwang and Lee 2007). Thus, NKA is considered a good biomarker of osmoregulation in teleosts. Several recent studies have reported that NKA activity changes with environment salinity variation (Fuentes et al. 1997; Laiz-Carrion et al. 2005; Malakpour Kolbadinezhad et al. 2012; Fisher et al. 2013; Handeland et al. 2014; Imsland et al. 2014; Vargas-Chacoff et al. 2014). Previous studies indicated that transferring fish to different salinities causes changes in oxygen consumption. The oxygen consumption of the *Mozambique tilapiaOreochromis mossambicus* enhances when salinity is increased (Zikos et al. 2014). Cao and Wang (2015) also found that the oxygen consumption of the mudskipper *Boleophthalmus pectinirostris* increases significantly when the salinity is increased from 12 to 27. Similar results were obtained in the inanga *Galaxias maculatus* (Urbina and Glover 2015). However, previous studies obtained different results possibly because of differences in species, acclimation duration, experimental design and measurement methodology. Morgan and Iwama (1991) summarised five oxygen consumption rate patterns from previous studies: (1) no change occurs in the oxygen consumption rate; (2) the oxygen consumption rate is minimum in isotonic salinity but increases in different salinities; (3) a linear relationship exists between the oxygen consumption rate and fluctuant salinity; (4) the oxygen consumption rate increases in hypotonic water and decreases under isotonic salinity condition; and (5) the highest oxygen consumption occurs in hypertonic water. Moreover, the relationship of salinity to the immune response of teleosts has received considerable attention in recent years (Harris and Bird 2000; Zhang et al. 2011; Arnason et al. 2013; Choi et al. 2013). Superoxide dismutase (SOD) is a common antioxidant enzyme that can protect organisms against reactive oxygen species-induced damage, which may lead to many disorders (Stadtman and Levine 2003; Seifried et al. 2007). Therefore, the antioxidant status in fish can be accurately reflected by SOD activity. Ma et al. (2014) indicated that salinity regulates the antioxidant activities of the juvenile golden pompano *Trachinotus ovatus*. They found that the SOD activity of this species is low at 10 ‰ salinity than at higher salinity levels. Acid phosphatase (ACP) may also act as an antioxidant that inhibits membrane nicotinamide adenine dinucleotide phosphate (NADPH) oxidase activity and consequently suppresses H_2_O_2_ and O_2_ production by immune cells (Glew et al. 1988). In most animal cells, alkaline phosphatase (ALP) is an important non-specific phospho-monoesterase enzyme that functions in phosphate metabolism. In aquatic organisms, responses to salinity include changes in oxygen consumption (Viarengo and Nott 1993) and osmoregulation (Lovett et al. 1994). Currently, several studies have focused on the effect of salinity changes on teleost osmoregulation, oxygen consumption rate and immunity response. These studies are mostly limited to marine or estuarine fish. Hence, the effects of salinity on ornamental freshwater fish remain unknown to date.

In many ornamental fish markets in China, some aquaculturists usually add salt to water during transportation and water renewal to maintain freshwater ornamental fish in a good shape (i.e. fish are more active and bright-coloured). However, the physiological effects of increased salinity on freshwater ornamental fish are unclear. Blood parrot, commonly known as bloody parrot or blood parrotfish, is a popular ornamental freshwater fish worldwide. Blood parrot is a man-made cross-bred fish hybridised from male *Cichlasoma citrinellum* and female *Cichlasoma synspilum* in Taiwan during the late 1980s and enjoyed in many countries, such as China and Japan, in recent years because of its bright red appearance and plump body. To explain the above phenomenon and explore whether increasing salinity favours the culture or transportation in freshwater ornamental fish, we chose blood parrotfish as a model to clarify the physiological mechanism based on the following hypotheses: (1) Increased water salinity saves energy for oxygen consumption by regulating NKA activity; (2) Increased water salinity stimulates fish immune responses by increasing antioxidant enzymes; (3) Increased water salinity helps preserve the fish skin pigment. Thus, the oxygen consumption, NKA activity, serum osmolality, immune-related enzyme activities in the gill, and skin carotenoid content of blood parrotfish were investigated by transferring fish from freshwater to water with four salinity levels (0.16, 2.5, 5 and 7.5 ‰) for 168 h, and physiological parameters were evaluated at 0, 6, 12, 24 and 168 h. Our results may also provide some useful information for freshwater ornamental fish production and logistics.

## Methods

### Animals and sampling methods

Blood parrots *C. synspilum* ♀ × *C. citrinellum* ♂ (total length 12–14 cm, body weight 52.5–54.0 g) were originally obtained from a commercial fish farm (Jiaxing, Zhejiang, China). All fish were maintained in a freshwater (a salinity of 0.16) recirculating tank with a 12L:12D photoperiod at 28 ± 1 °C in the Aquarium of Shanghai Ocean University, Shanghai, China. The treated salt water was prepared by adding artificial sea salt to freshwater. Blood parrots were transferred directly from freshwater to treated water with different salinity levels (0.16 as control, 2.5, 5 and 7.5) at the same time by nylon-net capture. Each treatment included three tanks (50 L) as three replicates with 25 fish each tank. During the experimental period (0–168 h), fish were reared in the experimental tanks without feeding. The fish from all groups were sampled at 0, 6, 12, 24 and 168 h at each sampling time point. Five individuals were randomly selected from each tank. Fish were anaesthetised with ice and killed immediately. Blood was collected via caudal puncture using a non-heparinised 2 mL syringe and then transferred to a 1.5 mL tube on ice. Blood samples were stored at 4 °C overnight, centrifuged at 800×*g* for 5 min and then serum was stored at −80 °C. The gills were removed and weighed.

### Sample processing

The tissue was homogenised in homogenisation solution (100 mM imidazole–HCl buffer, pH 7.0, 5 mM Na_2_ EDTA, 200 mM sucrose and 0.1 % sodium deoxycholate) with a motorised Teflon pestle at 600×*g* for 20 strokes on ice. After centrifugation (12,000×*g* for 30 min at 4 °C), the supernatant was stored at −80 °C until assay. Carotenoids were obtained from freeze dried skin in accordance with the method of Boonyaratpalin et al. (2001).

### Serum osmolality

Serum osmolality (mOsm/kg) was measured using a Vapro©Model 5520 vapour pressure osmometre (Wescor Inc., Logan, Utah, USA) from 10 μL of serum. Each sample was measured in duplicate.

### Serum ACP, ALP, SOD and gill NKA activity assay

The activities of ACP, ALP, SOD and NKA were determined using commercial kits (Nanjing Jiancheng Bioengineering Institute, Nanjing, China) in accordance with the method of Ma et al. (2014). (1) ACP and ALP activities were measured by using disodium phenylphosphate as the substrate. The enzyme unit definitions of ACP (U/100 mL serum) and ALP (U/100 mL serum) were expressed as the degradation of 1 mg phenol/mg serum at 37 °C within 30 and 15 min, respectively. (2) SOD activity was assayed using the xanthine/xanthine oxidase method based on the production of O_2_^−^ anions. (3) NKA activity was measured using an endpoint phosphate ATP hydrolysis protocol following the kit. The inorganic phosphate released was determined by colorimetric assays, and NKA activity was expressed as micromole inorganic phosphate per mg protein per hour.

### Carotenoid content

Carotenoid contents were determined as described by Boonyaratpalin et al. (2001). The absorption of solution was read at 470 nm. The carotenoid content was calculated in accordance with the formula:

$$ {\text{S}} = \left( {{\text{A}} \times {\text{K}} \times {\text{V}}} \right)/\left( {{\text{E}} \times {\text{G}}} \right), $$

where S is the carotenoid content (mg/kg), A is the absorbance, K is a constant (10^4^), V is the volume of extracting solution (mL), E is the extinction coefficient (2500) and G is the sample weight (g).

### Oxygen consumption rate

The oxygen consumption rates of blood parrots under different salinity levels were determined using computerised, intermittent-flow respirometry (LoligoSystems, Hobro, Denmark, Beauregard et al. 2013). The system consisted of four glass chambers (180 mm long, 62 mm inner diameter; 0.54 L) outfitted with fibre optic oxygen probes (OXY-4 mini, PreSens, Regensburg, Germany) immersed in a 120 L tank of aerated treated water maintained at 28 °C. The fish were placed in the chambers and left to acclimatise for 4 h until oxygen consumption of the test fish reached a steady state level. The change in oxygen concentration (α) for each chamber was calculated as slope (△O_2_saturation/△t), and the oxygen consumption rate (*M*O_2_; mg O_2_/kg h) for each fish was calculated by the formula:

$$ M{\text{O}}_{2} =\upalpha \times {\text{Vresp}} \times\upbeta \times {\text{Mb}}^{ - 1} , $$

where α is the oxygen concentration, Vresp is the volume of each glass chamber minus the volume of the fish (L), β is the oxygen solubility (adjusted nightly for both temperature and barometric pressure) and Mb is the fish mass (kg) prior to placing in a respirometre chamber. During each trial, the coefficient of determination (r^2^) for all slope measurements was >0.95, and the oxygen concentration in each chamber was recorded every 2 s. Experiments were designed such that the oxygen consumption in each individual chamber was quantified with 10 min cycles consisting of a measurement phase (5 min), a flushing period (4 min) to replace water in each chamber and a waiting period (1 min) following each flushing prior to commencing measurements. During each measurement period, water from the chambers was continually recirculated across the fibre optic oxygen probes to ensure adequate mixing, and all calculated dissolved oxygen values were corrected for background oxygen consumptions generated for each specific fish and chamber prior to commencing experiments. The fibre optic oxygen probes were calibrated with oxygen-free water and fully aerated water regularly throughout the experiments, and data were recorded with AutoResp software (version 2.0.1; Loligo Systems, Tjele, Denmark).

### Statistical analysis

Prior to the analysis, normality of the data was evaluated by using the Shapiro–Wilk’s test, and homogeneity of variances was checked by Levene’s test using the statistical software SPSS 17.0. One-way ANOVA was applied to evaluate the effects of salinity on all parameters at each time point, and Student–Newman–Keuls tests were performed to determine which salinity treatments were different. For the time effects, paired *t* test was used to compare the difference between each sampling time and 0 h at each salinity treatment, respectively. Differences were considered significant at *P* < 0.05. The results are expressed as mean ± SD.

## Results

### Enzymatic activities

**Fig. 1:**
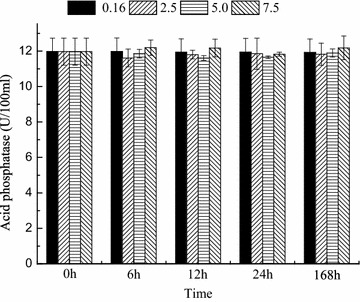
Acid phosphatase activity over time in blood parrotfish serum following transfer to various salinities

**Fig. 2:**
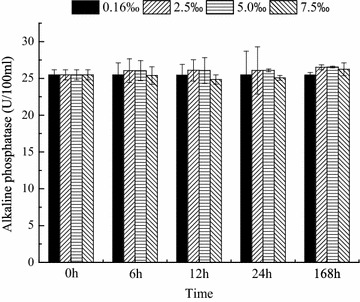
Alkaline phosphatase activity over time in blood parrotfish serum following transfer to various salinities

**Fig. 3:**
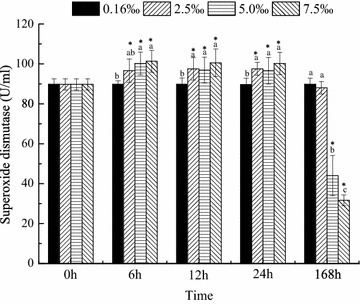
Superoxide dismutase over time in blood parrotfish serum following transfer to various salinities. *Different small letters* indicate significant differences between different salinities at a fixed time, whereas *asterisk* indicates significant differences between initial moment (0 h) and other sampling times within a given salinity, respectively

**Fig. 4:**
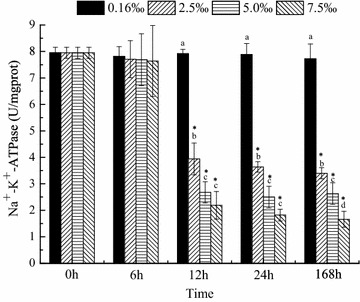
Na^+^–K^+^-ATPase activity over time in blood parrotfish gill following transfer to various salinities. *Different small letters* indicate significant differences between different salinities at a fixed time, whereas *asterisk* indicates significant differences between initial moment (0 h) and other sampling times within a given salinity, respectively

The activities of ACP, ALP, SOD and NKA were measured at 0, 6, 12, 24 and 168 h. The activities of ACP and ALP were maintained at normal levels regardless of the salinity level and time (Figs. 1, 2). SOD activity was significantly affected by salinity and time (*P* < 0.05, Fig. 3). Compared with that in the control group, the SOD activities in the trial groups increased within 6 h and then maintained at a high level until 24 h. On day 7, the SOD activities in all trial groups decreased; the SOD activity in the salinity 2.5 ‰ group recovered to the same level as that in the control group. Nevertheless, the SOD activities in the salinity 5 and 7.5 ‰ groups were significantly lower (*P* < 0.05) than those in the control group (Fig. 3). NKA activity was significantly affected by salinity and time (Fig. 4). After 12 h, NKA activity significantly decreased with increasing salinity level and showed the lowest value at 7.5 ‰. At high salinity levels, NKA activity decreased with time and showed the lowest value at 168 h (Fig. 4).

### Serum osmolality

**Fig. 5:**
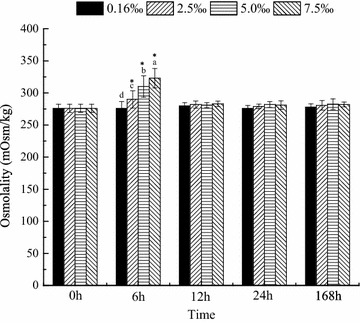
Osmolality over time in blood parrotfish serum following transfer to various salinities. *Different small letters* indicate significant differences between different salinities at a fixed time, whereas *asterisk* indicates significant differences between initial moment (0 h) and other sampling times within a given salinity, respectively

Serum osmolality was significantly affected by salinity and time. The serum osmolality in the control group was similar at all sampling times, whereas that in all trial groups showed peaks at 6 h. Significant differences in serum osmolality were observed between various salinity groups (7.5 > 5 > 2.5 > 0.16 ‰, *P* < 0.05). Thereafter, the serum osmolality in all trial groups returned to the level of the control group within 12 h and remained stable until the end of the experiment (Fig. 5).

### Oxygen consumption rate and carotenoid content

**Fig. 6:**
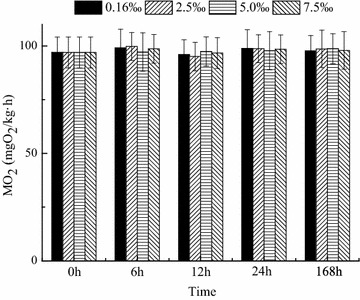
Oxygen consumption rate over time in blood parrotfish following transfer to various salinities

**Fig. 7:**
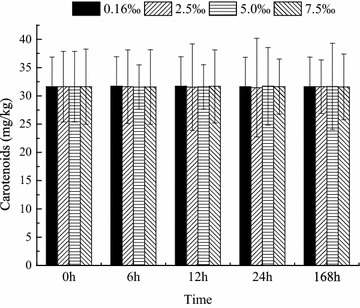
Carotenoid contents over time in blood parrotfish skin following transfer to various salinities

The oxygen consumption rate was not significantly affected by salinity and time (*P* > 0.05). No significant interaction was observed between salinity and time. The *M*O_2_ in all groups was stable during the experimental period (Fig. 6). The carotenoid content in the skin of the fish was unaffected by salinity and time (*P* > 0.05, Fig. 7).

## Discussion

Compared with that in the control group, the SOD activities in all trial groups increased after 6 h of exposure, suggesting that increased salinity can stimulate SOD activity within a short period. Nevertheless, high SOD activities were not maintained at all sampling times. The value changed greatly at 168 h. As shown in Fig. 3, SOD activity returned near the initial level in the salinity 2.5 ‰ group, whereas those in the salinity 5 and 7.5 ‰ groups reduced significantly by 50 and 65 %, respectively. The study on the juvenile silver pomfret *Pampus argenteus* by Yin et al. (2011) showed that salinity change might stimulate SOD activity to some extent, but the activity would recover more or less with the elongation of time. Similar results were also found in pompano. Liu et al. (2013) indicated that increased salinity enhances liver SOD enzyme activity. In accordance with the results of our research, adding appropriate salt to water could temporarily enhance the antioxidant ability of fish. However, this strategy is insufficient or harmful for long-term culture.

Phosphatases remove phosphate groups from their substrates by hydrolysing phosphoric acid monoesters into phosphate ions and molecules with free hydroxyl groups. ACP and ALP are important phosphatases in aquatic organisms; these enzymes participate in the degradation of foreign proteins, carbohydrates and lipids (Liu et al. 2004). ACP is a typical lysosome enzyme that plays a role in killing and digesting pathogens in immune responses (Yin et al. 2014). ALP is a multi-functional enzyme involved in immune responses (Xing et al. 2002). Both enzymes are sensitive to environment change. In the present study, ACP activity did not change significantly, indicating that increased salinity levels only slightly affected the physiological functions of blood parrotfish. The result is supported by Fang et al. (2014), who found that the ACP activities in the gill and kidney of the juvenile tongue sole Cynoglossus semilaevis show no significant difference between low salinity and high salinity treatments. Similar to ACP activities, ALP activities were also not affected by increased salinity. However, the ALP activity in the serum of the cobia *Rachycentron canadum* increases when the salinity is within the range of 5 to 37 (Feng et al. 2007). In general, ACP and ALP activities were not significantly altered. This result indicates that blood parrotfish can easily adapt to salinity increase.

Among the transporters that modulate ion fluxes, NKA actively transports Na^+^ out and K^+^ in animal cells (Post and Jolly 1957). In addition, NKA is generally involved in the maintenance of internal hypo-osmotic state when the environmental salinity changes. Changes in environmental salinity are usually accompanied by changes in NKA activity (Marshall 2002; Hirose et al. 2003; Burg et al. 2007). In this study, the NKA activity in fish was affected significantly by salinity. NKA activity decreased with increasing salinity. NKA activity was significantly lower in the 7.5 ‰ group than in the other groups after 168 h of exposure (*P* < 0.05). Meanwhile, osmolality increased with increasing salinity (Table 1), but the serum osmolality of blood parrotfish was not altered (Fig. 5). The result is consistent with a previous study that pointed out that Atlantic sturgeon could regulate blood plasma osmolality at similar levels regardless of salinity (Martinez-Alvarez et al. 2002). Therefore, blood parrotfish can move along a salinity gradient. In addition, the gradients between medium and body fluid became small as the salinity level increased. Thus, energy expenditure on osmoregulation decreased. However, in the present study, the oxygen consumption rate did not change in the various groups during the experiment. The result is consistent with the findings of other studies (Swanson 1998; Haney et al. 1999; Sardella and Brauner 2008), which indicates no clear trend of lowest oxygen uptake at either normal lifecycle salinity or other salinities. As summarised by Bœuf and Payan (2001), 20–68 % of the total energy expenditure is estimated to be consumed by osmoregulation in different species. This condition implies that although the total energy expenditure of the fish does not change, the allocation of energy is altered clearly. Morgan and Iwama (1991) stated that estimates of osmoregulation costs based on whole-fish oxygen consumption should consider the effects of other metabolic processes that respond to salinity changes. During salinity adaptation, several hormones affect different pathways of energetic metabolism (Polakof et al. 2006; Sangiao-Alvarellos et al. 2007), and other organs (e.g. the brain, liver and kidney) also show changes in energetic metabolism (Sangiao-Alvarellos et al. 2005, 2006). Sangiao-Alvarellos et al. (2003) indicated that acclimation of *Sparus aurata* to various salinities alters the energy metabolism of osmoregulatory and non-osmoregulatory organs. In the gills, NKA activity improved, the capacity for use of exogenous glucose and the pentose phosphate pathway decreased, and glycolytic potential increased with increasing salinity. In the brain, freshwater-acclimated fish displayed enhanced potentials for glycogenolysis, use of exogenous glucose and glycolysis compared with seawater-acclimated fish (Sangiao-Alvarellos et al. 2003). Furthermore, the levels of lactate and ATP in the brain decreased with increasing salinity (Sangiao-Alvarellos et al. 2003). However, we did not assess the parameters related in non-osmoregulatory organs in the present experiment.

**Table 1:** Salinity and osmolality (mean ± SD, n = 5) of trial water during the experiment period

Salinity	0.16 ± 0.01	2.5 ± 0.05	5 ± 0.06	7.5 ± 0.05
Osmolality (mOsm/kg)	21 ± 1	82.5 ± 1.1	251 ± 1.2	210 ± 1.5

The carotenoid content of the skin in some ornamental fish is crucial because it would affect acceptability by consumers. In a recent study, Eslamloo et al. (2015) have stated that background colour could affect goldfish skin pigmentation. The carotenoid concentration in the skin significantly decreases in white background in comparison with the other groups. Doolan et al. (2008) recommended that holding snapper in white cages at high densities greatly improves skin lightness in comparison with black cages. In the present study, the carotenoid contents in the blood parrotfish skin did not change in the various salinity groups. This result implies that salinity change could not affect the skin pigmentation of blood parrotfish.

## Conclusions

On the basis of the estimated parameters, adding appropriate salt into water provides benefits to the transportation or short-term culture of blood parrotfish by temporarily elevating the antioxidant ability of this ornamental fish. However, this strategy is insufficient for long-term culture.

## References

[CR1] Arnason T, Magnadottir B, Bjornsson B, Steinarsson A, Bjornsson BT (2013). Effects of salinity and temperature on growth, plasma ions, cortisol and immune parameters of juvenile Atlantic cod (*Gadus morhua*). Aquaculture.

[CR2] Beauregard D, Enders E, Boisclair D, Kidd K (2013). Consequences of circadian fluctuations in water temperature on the standard metabolic rate of Atlantic salmon parr (*Salmo salar*). Can J Fish Aquat Sci.

[CR3] Bœuf G, Payan P (2001). How should salinity influence fish growth?. Comp Biochem Physiol C Toxicol Pharmacol.

[CR4] Boonyaratpalin M, Thongrod S, Supamattaya K, Britton G, Schlipalius LE (2001). Effects of β-carotene source, *Dunaliella salina*, and astaxanthin on pigmentation, growth, survival and health of *Penaeus monodon*. Aquac Res.

[CR5] Burg MB, Ferraris JD, Dmitrieva NI (2007). Cellular response to hyperosmotic stresses. Physiol Rev.

[CR6] Cao F, Wang H (2015). Effects of salinity and body mass on oxygen consumption and ammonia excretion of *mudskipper Boleophthalmus pectinirostris*. Chin J Oceanol Limnol.

[CR7] Choi K, Cope WG, Harms CA, Law JM (2013). Rapid decreases in salinity, but not increases, lead to immune dysregulation in *Nile tilapia*, *Oreochromis niloticus* (L.). J Fish Dis.

[CR8] Doolan BJ, Allan GL, Booth MA, Jones PL (2008). Effects of cage netting colour and density on the skin pigmentation and stress response of Australian snapper *Pagrus auratus* (Bloch & Schneider, 1801). Aquac Res.

[CR9] Eslamloo K, Akhavan SR, Eslamifar A, Henry MA (2015). Effects of background colour on growth performance, skin pigmentation, physiological condition and innate immune responses of goldfish, *Carassius auratus*. Aquac Res.

[CR10] Fang ZH, Tian XL, Dong SL, Dai C, Wang GD (2014). Effects of salinity on the activity of non-specific immune enzymes of juvenile tongue sole cultured in various salinities. J Ocean Univ China.

[CR11] Feng J, Xu LW, Lin HZ, Guo ZX, Guo GX (2007). Effects of salinity on growth and several immune parameters of juvenile cobia, *Rachycentron canadum*. J Fish Sci China.

[CR12] Fisher C, Bodinier C, Kuhl A, Green C (2013). Effects of potassium ion supplementation on survival and ion regulation in Gulf killifish *Fundulus grandis* larvae reared in ion deficient saline waters. Comp Biochem Physiol A Mol Integr Physiol.

[CR13] Fuentes J, Soengas JL, Rey P, Rebolledo E (1997). Progressive transfer to seawater enhances intestinal and branchial Na^+^–K^+^-ATPase activity in non-anadromous rainbow trout. Aquac Int.

[CR14] Glew RH, Saha AK, Das S, Remaley AT (1988). Biochemistry of the leishmania species. Microbiol Rev.

[CR15] Handeland SO, Imsland AK, Nilsen TO, Ebbesson LO, Hosfeld CD, Pedrosa C, Toften H, Stefansson SO (2014). Osmoregulation in Atlantic salmon *Salmo salar* smolts transferred to seawater at different temperatures. J Fish Biol.

[CR16] Haney DC, Nordlie FG, Binello J (1999). Influence of simulated tidal changes in ambient salinity on routine metabolic rate in *Cyprinodon variegatus*. Copeia.

[CR17] Harris J, Bird DJ (2000). Modulation of the fish immune system by hormones. Vet Immunol Immunopathol.

[CR18] Hirose S, Kaneko T, Naito N, Takei Y (2003). Molecular biology of major components of chloride cells. Comp Biochem Physiol B Biochem Mol Biol.

[CR19] Hwang PP, Lee TH (2007). New insights into fish ion regulation and mitochondrion-rich cells. Comp Biochem Physiol A Mol Integr Physiol.

[CR20] Imsland AK, Handeland SO, Stefansson SO (2014). Photoperiod and temperature effects on growth and maturation of pre- and post-smolt Atlantic salmon. Aquac Int.

[CR21] Laiz-Carrion R, Guerreiro PM, Fuentes J, Canario AV, Martin Del Rio MP, Mancera JM (2005). Branchial osmoregulatory response to salinity in the gilthead sea bream, *Sparus auratus*. J Exp Zool A Comp Exp Biol.

[CR22] Liu S, Jiang X, Hu X, Gong J, Hwang H, Mai K (2004). Effects of temperature on non-specific immune parameters in two scallop species: *Argopecten**irradians* (Lamarck 1819) and *Chlamys farreri* (Jones & Preston 1904). Aquac Res.

[CR23] Liu R, Ou Y, Li J, Su H, Cao S, Wang Y (2013). Effects of salinity and temperature on the activity of antioxidant enzymes in livers of selective group of *Trachinotus ovatus*. Chin J Zool.

[CR24] Lovett DL, Towle DW, Faris JE (1994). Salinity-sensitive alkaline phosphatase activity in gills of the blue crab, *Callinectes sapidus*, *rathbun*. Comp Biochem Physiol B Comp Biochem.

[CR25] Ma ZH, Zheng PL, Guo HY, Jiang SG, Qin JG, Zhang DC, Liu XL (2014). Salinity regulates antioxidant enzyme and Na^+^–K^+^-ATPase activities of juvenile golden pompano *Trachinotus ovatus* (*Linnaeus 1758*). Aquac Res.

[CR26] Malakpour Kolbadinezhad S, Hajimoradloo A, Ghorbani R, Joshaghani H, Wilson JM (2012). Effects of gradual salinity increase on osmoregulation in Caspian roach *Rutilus caspicus*. J Fish Biol.

[CR27] Marshall WS (2002). Na^+^, Cl^−^, Ca^2+^ and Zn^2+^ transport by fish gills: retrospective review and prospective synthesis. J Exp Zool.

[CR28] Marshall W, Bryson S (1998). Transport mechanisms of seawater teleost chloride cells: an inclusive model of a multifunctional cell. Comp Biochem Physiol A Mol Integr Physiol.

[CR29] Martinez-Alvarez R, Hidalgo M, Domezain A, Morales A, García-Gallego M, Sanz A (2002). Physiological changes of *sturgeon Acipenser naccarii* caused by increasing environmental salinity. J Exp Biol.

[CR30] Morgan JD, Iwama GK (1991). Effects of salinity on growth, metabolism, and ion regulation in juvenile rainbow and steelhead trout (*Oncorhynchus mykiss*) and fall chinook salmon (*Oncorhynchus tshawytscha*). Can J Fish Aquat Sci.

[CR31] Polakof S, Arjona FJ, Sangiao-Alvarellos S, Martin del Rio MP, Mancera JM, Soengas JL (2006). Food deprivation alters osmoregulatory and metabolic responses to salinity acclimation in gilthead sea bream *Sparus auratus*. J Comp Physiol B.

[CR32] Post RL, Jolly PC (1957). The linkage of sodium, potassium, and ammonium active transport across the human erythrocyte membrane. Biochim Biophys Acta.

[CR33] Sangiao-Alvarellos S, Laiz-Carrión R, Guzmán JM, del Río MPM, Miguez JM, Mancera JM, Soengas JL (2003). Acclimation of *S. aurata* to various salinities alters energy metabolism of osmoregulatory and nonosmoregulatory organs. Am J Physiol Regul Integr Comp Physiol.

[CR34] Sangiao-Alvarellos S, Arjona FJ, Martin del Rio MP, Miguez JM, Mancera JM, Soengas JL (2005). Time course of osmoregulatory and metabolic changes during osmotic acclimation in *Sparus auratus*. J Exp Biol.

[CR35] Sangiao-Alvarellos S, Polakof S, Arjona FJ, García-López A, Martín del Río MP, Martínez-Rodríguez G, Míguez JM, Mancera JM, Soengas JL (2006). Influence of testosterone administration on osmoregulation and energy metabolism of gilthead sea bream *Sparus auratus*. Gen Comp Endocrinol.

[CR36] Sangiao-Alvarellos S, Míguez JM, Soengas JL (2007). Melatonin treatment affects the osmoregulatory capacity of rainbow trout. Aquac Res.

[CR37] Sardella BA, Brauner CJ (2008). The effect of elevated salinity on ‘California’ *Mozambique tilapia* (*Oreochromis mossambicus* × *O. urolepis hornorum*) metabolism. Comp Biochem Physiol C Toxicol Pharmacol.

[CR39] Seifried HE, Anderson DE, Fisher EI, Milner JA (2007). A review of the interaction among dietary antioxidants and reactive oxygen species. J Nutr Biochem.

[CR40] Stadtman ER, Levine RL (2003). Free radical-mediated oxidation of free amino acids and amino acid residues in proteins. Amino Acids.

[CR41] Swanson C (1998). Interactive effects of salinity on metabolic rate, activity, growth and osmoregulation in the euryhaline milkfish (*Chanos chanos*). J Exp Biol.

[CR42] Urbina MA, Glover CN (2015). Effect of salinity on osmoregulation, metabolism and nitrogen excretion in the amphidromous fish, inanga (*Galaxias maculatus*). J Exp Mar Biol Ecol.

[CR43] Vargas-Chacoff L, Moneva F, Oyarzun R, Martinez D, Munoz JLP, Bertran C, Mancera JM (2014). Environmental salinity-modified osmoregulatory response in the sub-Antarctic notothenioid fish *Eleginops maclovinus*. Polar Biol.

[CR44] Viarengo A, Nott JA (1993). Mechanisms of heavy metal cation homeostasis in marine invertebrates. Comp Biochem Physiol C Comp Pharmacol.

[CR45] Xing J, Zhan WB, Zhou L (2002). Endoenzymes associated with haemocyte types in the scallop (*Chlamys farreri*). Fish Shellfish Immunol.

[CR46] Yin F, Peng S, Sun P, Shi Z (2011). Effects of low salinity on antioxidant enzymes activities in kidney and muscle of juvenile silver pomfret *Pampus argenteus*. Acta Ecol Sin.

[CR47] Yin F, Dan XM, Sun P, Shi ZH, Gao QX, Peng SM, Li AX (2014). Growth, feed intake and immune responses of orange-spotted grouper (*Epinephelus coioides*) exposed to low infectious doses of ectoparasite (*Cryptocaryon irritans*). Fish Shellfish Immunol.

[CR48] Zhang YJ, Mai KS, Ma HM, Ai Q, Zhang W, Xu W (2011). Rearing in intermediate salinity enhances immunity and disease-resistance of turbot (*Scophthalmus maximus L.*). Acta Oceanol Sin.

[CR49] Zikos A, Seale AP, Lerner DT, Grau EG, Korsmeyer KE (2014). Effects of salinity on metabolic rate and branchial expression of genes involved in ion transport and metabolism in *Mozambique tilapia* (*Oreochromis mossambicus*). Comp Biochem Physiol A Mol Integr Physiol.

